# Simulation of emergency evacuation from construction site of prefabricated buildings

**DOI:** 10.1038/s41598-022-06211-w

**Published:** 2022-02-17

**Authors:** Mufeng Xiao, Xihua Zhou, Xinxin Pan, Yanan Wang, Jue Wang, Xianlin Li, Yashengnan Sun, Yumeng Wang

**Affiliations:** 1 College of Safety Science & Engineering, Liaoning Technical University, Fuxin, 123000 Liaoning China; 2 Key Laboratory of Mine Thermodynamic Disasters & Control of Ministry of Education, Liaoning Technical University, Fuxin, 123000 Liaoning China; 3 College of Architecture and Transportation, Liaoning Technical University, Fuxin, 123000 Liaoning China

**Keywords:** Energy science and technology, Engineering

## Abstract

To ensure the safe construction of prefabricated buildings and improve the efficiency of the safe evacuation of construction personnel after a fire caused by improper operation during construction, this study used the PyroSim software to numerically simulate a fire situation based on the size and volume of a prefabricated building construction site. The variation rules of smoke visibility, CO concentration, and ambient temperature in the construction site of prefabricated buildings were analyzed and the available safe evacuation time was determined. Moreover, the Pathfinder software was used for simulation in combination with the physical attributes of personnel, evacuation speed, and personnel proportions. The time required for safe evacuation was determined and the factors influencing the evacuation time, such as the quantity and location of stacked prefabricated components, machinery, and appliances, and the number of on-site construction personnel, were analyzed. The data collected by the temperature sensor, CO concentration sensor, and visibility sensor reveal that the visibility and crash time are the key factors restricting the efficiency of personnel avoidance and evacuation. At 400 s, the visibility at the escape exit of the prefabricated apartment construction site was lower than 5 m. The crashing time of the building was 360 s, which is the critical point for casualties. The first emergency evacuation simulation took 398.7 s. The required safe evacuation time (T_REST_) > available safe evacuation time (T_ASET_), and the original site layout cannot facilitate the safe evacuation of all construction workers. The evacuation time can be effectively reduced by re-planning the stacking positions of prefabricated construction site components, construction equipment, and other items, and reducing the number of personnel in the construction plane. The results of the second simulation reveal that the safe evacuation time (T_REST_) is 355.2 s. Because it is required that the safety evacuation time (T_REST_) < available safe evacuation time (T_ASET_), the results are in line with the emergency evacuation requirements. The findings of this study can provide a theoretical basis for the rational planning of evacuation passages at the construction sites of prefabricated buildings and assist the management of construction site safety.

## Introduction

With the rapid development of China’s economy, the level of urbanization is further accelerating and prefabricated construction is rapidly developing owing to its advantages of low carbon emissions, environmental efficiency, high technological content, and management innovation. When COVID-19 broke out in China in 2020, Huoshenshan Hospital and Leishenshan Hospital were built in only 10 days. The high efficiency, low cost, and reduced pollution advantages of prefabricated buildings have resulted in the construction market fully realizing the efficiency and advantages of prefabricated building construction in emergency situations. However, owing to the frequency of accidents in the construction industry, the safety of human lives and property are of great concern, and more attention is being directed toward the safe evacuation of construction personnel from assembly construction sites, which is gradually becoming a hot research topic domestically and abroad. Wu^1^ used the Pathfinder software to simulate the evacuation process in an office building construction site, and optimized the construction schedule and site layout using the evacuation simulation results in combination with various rules. Wang et al.^2^ used the PyroSim software to numerically simulate a fire situation, and analyzed the change rules of smoke visibility and environmental temperature at each stair exit of the office building when the fire occurs under the failure of the automatic fire extinguishing system, fire door failure, and so on. Moreover, they conducted evacuation simulation using the Pathfinder software. The influence of the total number of evacuees, environmental temperature, and environmental visibility on the evacuation efficiency was analyzed. Wahyu Sujatmiko et al.^3^ used the FDS and EVAC software to simulate the emergency evacuation of a high-rise residential construction project, analyzed the spreading law of smoke based on the principle of fluid dynamics, and assessed the safety of the emergency evacuation of construction personnel from high-rise buildings. Ruggiero et al.^4^ experimentally analyzed the influence of people’s behavioral habits on the evacuation process, and compared the differences between the experimental data and the simulation results obtained for the evacuation behavioral rules using the FDS and EVAC software. Helbing^5^ used the social force model to simulate the evacuation process in the case of fire, and set up traffic signs at particular positions inside the building to guide the evacuation under emergency conditions. Nishinari et al.^6^ used a CA model to simulate a safe personnel evacuation, analyzed the positions of obstacles in the building, and optimized the site layout in accordance with the analysis results. Yu^7^ used the Pathfinder software and considered different structural units to conduct a simulation and assess the impact of the room entrance location, length of the evacuation time, and impact of the evacuation efficiency. The effects of the width, angle, quantity, and total width on the evacuation were investigated by building the structural models of corridor exits with different crowd densities. Liu^8^ used the Pathfinder software to simulate the safe emergency evacuation of primary school personnel and comprehensively analyzed a school building. They analyzed the influence of traffic space design, personnel behavior, psychological fluctuations, and other factors on the evacuation time under fire conditions, and proposed optimization measures for the shortcomings of existing programs. Xie et al.^9^ used PyroSim and FDS to model and numerically simulate a fire situation, and analyzed the temperature, toxic and harmful gases, and visibility to determine T_ASET._ Moreover, an evacuation simulation was conducted using Pathfinder to determine the required evacuation time and analyze the congestion problems during the evacuation process. Sun et al.^10^ jointly used the PyroSim and Pathfinder software to numerically simulate the spread of fire and developed rules for different emergency evacuation situations. A model was established to simulate the fire and evacuation of typical school buildings. According to the results and data obtained from the simulation, the change laws of the spread of fire, smoke diffusion, toxic gas concentration, temperature distribution, and visibility, were analyzed. Many other studies have conducted simulations to investigate the organized evacuation of on-site construction personnel^11–20^.

Existing research does not consider the impact of smoke diffusion and reduced visibility during fire evacuations or the impact of personnel distribution on the evacuation. Additionally, research on evacuation simulation and emergency rescue route determination under extreme conditions is lacking. This study considered the case of fire on a prefabricated building construction site as the research object, and used the fire characteristics to carry out risk analysis for the prefabricated building construction site. Additionally, by investigating the fire development process and fire scenario design theory, a fire numerical simulation model was established for the prefabricated building construction site, and important fire scenarios and fire performance parameters were analyzed. This study conducted an experiment to study the evacuation of personnel from the prefabricated building construction site and determine the best escape route and escape time. The prefabricated building construction site fire scene was constructed using fire simulation methods. The law governing the spread of smoke through the building in the event of fire was analyzed, and the impact of smoke on the various properties of air and the evacuation of personnel was evaluated. According to the evacuation experiment for obtaining the personnel movement parameters during the evacuation process, the movement rules of the personnel were analyzed, the corresponding facilities of the building were reasonably arranged, and the evacuation capacity of the building itself was improved. The findings of this study can provide a theoretical basis and technical support for the implementation of self-rescue measures during the construction of a prefabricated building unit.

This study considered the dynamics of a prefabricated building construction site, and the construction phase was divided into the basic phase, main structure phase, and decoration phase according to the construction sequence, so as to facilitate the analysis of the project components prone to fire. In the renovation stage and completion acceptance stage, various fire factors should be comprehensively considered to avoid the limitations and one-sidedness of simulation, and multiple fire scenarios should be selected to analyze and compare the fire risks of buildings. According to research data, the main structure stage and the decoration stage are the main stages of fire, and the main combustibles are prefabricated wooden formwork and thermal insulation decoration materials. This study focused on the impact of the quantity and location of items and number of operators on the time needed for evacuation from the prefabricated building construction site. Owing to space limitations, this study considered the construction stage of the main structure as an example. The surrounding area of the room is prefabricated formwork made of wood material, which will burn during a fire.

**Figure 1:**
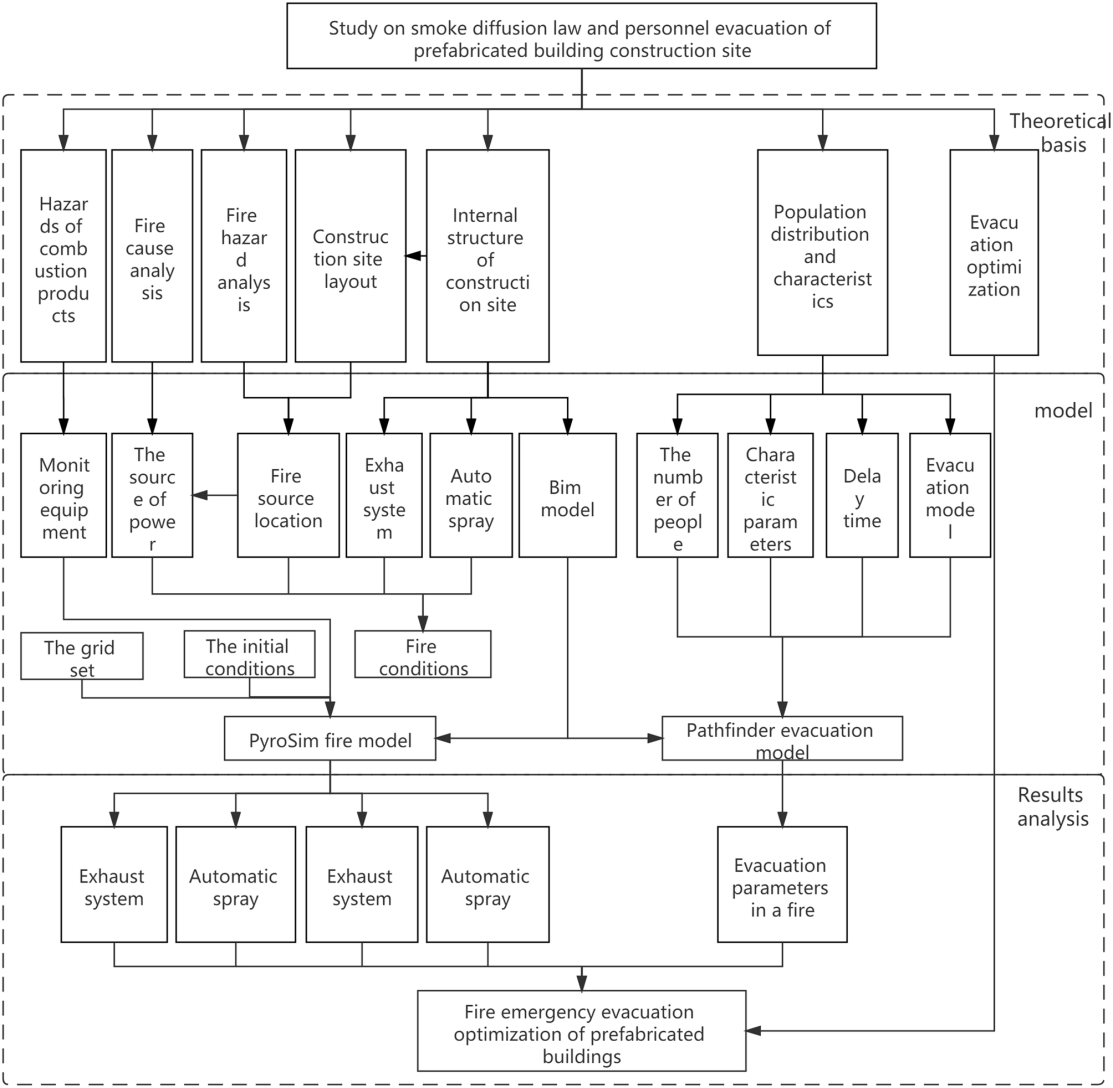
General framework for fire emergency evacuation from prefabricated building construction site.

From the perspective of a macro-evacuation strategy, this study considered a prefabricated apartment for talented people to investigate the evacuation of people from a prefabricated building construction site in the event of fire. The overall framework of fire emergency evacuation from the prefabricated building construction site is proposed as shown in Fig. 1. Building information modeling (BIM) technology was used to construct a parameterized information model, and the fire dynamics software PyroSim and Pathfinder were used to perform FDS smoke numerical simulation and emergency evacuation analysis for the prefabricated building construction site under fire conditions. First, the Revit software was used to establish a building information model, which was then imported into the PyroSim and Pathfinder simulation software for fire smoke analysis and personnel evacuation analysis. The PyroSim software was used to analyze the smoke layer height, smoke temperature, visibility, and CO concentration in different areas. The critical value of each factor was determined, and the safe evacuation time (T_ASET_) for personnel in different operating areas was obtained. Pathfinder software was used to simulate the time (T_RSET_) required for the safe evacuation of construction personnel in the event of fire on a prefabricated construction site, and the personnel safety was assessed. Finally, Pathfinder was used to further optimize the evacuation process such that the simulation results would more closely resemble the actual situation.

## Numerical modeling and fire simulation

### Introduction to PyroSim and simulated objects

To simulate the fire development process, it is necessary to select a suitable simulation model. Through the analysis of such models, an intuitive understanding of the fire spreading process can be gained, and the temperature field distribution, smoke distribution, and visibility distribution in the fire scene can be qualitatively and quantitatively analyzed^21^. Presently, the main fire simulation software are as follows: DETACT-QS, used to calculate the power of the fire source; FDS, FLUENT, and CFAST, used to calculate fire smoke distribution, and so on. The application of FLUENT and other software to fire simulations requires a strong fluid mechanics background, which renders them unsuitable for use in this study. The FDS software is specifically developed for fire simulations and is the most widely used fire simulation software owing to its ease of use.

The FDS software was developed by the Building Fire Laboratory of The National Institute of Technology and Standards to calculate the fire dynamics. The FDS software can be used to simulate and analyze the smoke spreading process^22^, carry out the geometric modeling of building structures, set the monitoring position of fire information such as the wall thermal conductivity parameters, fire type, smoke temperature, and visibility, and investigate the change law of the fire parameters during the combustion process. The simulation results are visualized by the Smoke View software, including the temperature, smoke concentration, smoke speed, and other parameters of plane distribution during the smoke spreading process. As a pre-processing software for FDS, PyroSim can be used to quickly build models, set the parameters, and adjust the monitoring point settings, in addition to other functions. PyroSim has the following characteristics and capabilities:

1. Hydrodynamic modeling. The Neville–Stokes equation can be used to quantitatively calculate thermally driven, low-velocity fluids.
2. Combustion modeling. Different combustion reactions can be set up and simulated by setting the combustion mixture.
3. Radiative transport. Radiative transport equations can be used to calculate the radiative transport.
4. Unique geometry. The software allows the setting of obstacles.
5. Boundary conditions. The thermal boundary conditions of the materials can be defined, and the combustion characteristics can be set.

Therefore, this study selected PyroSim to numerically simulate the fire scene of an assembly building construction site.

**Figure 2:**
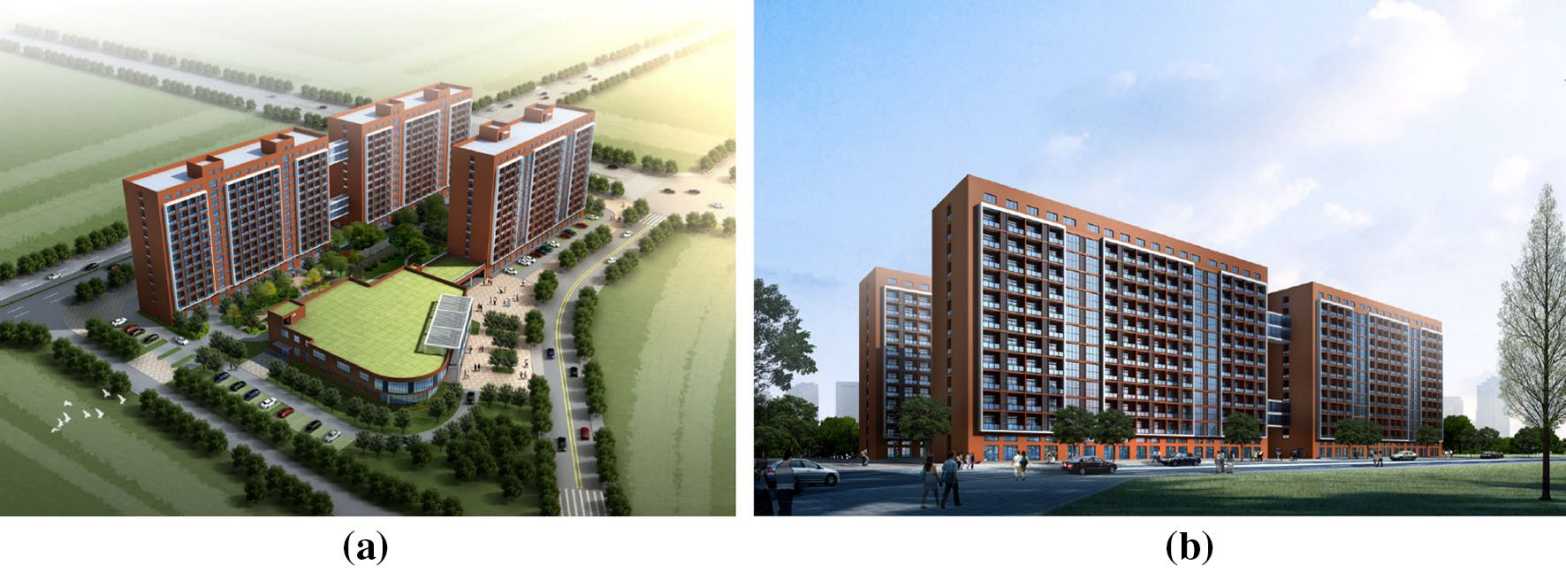
Architectural renderings of prefabricated apartment for talented people.

The prefabricated building talent apartment project considered in this study is designed to facilitate education and scientific research. The land area is 13,719.29 m^2^, the total construction area is 45,641.49 m^2^, the considered construction area is 3313.74 m^2^, the plot ratio is 4.31, and the building density is 24.15%. The project effect is shown in Fig. 2. The structural form of the project is a frame shear wall structure, and the assembled components are a composite floor slab, composite beam, external wall hanging plate, light partition wall, precast staircase, and so on, with a precast rate of 30.53%. After the installation of the precast components, the entire post-cast concrete is combined with the frame column beam and so on.

This study considered building #F02 of the Talent apartment complex as an example for establishing a safety information model. The talent apartment building consists of two high-rise buildings, the fire protection rating is One, and each floor has two evacuation stairs. The building has 13 floors and a total height of 40.95 m. The ground floor is equipped with a duty room, public laundry room, storage room, and so on. The 2nd to 13th floors consist of two-room student dormitories that can accommodate 720 students. The room space in a standard floor of the prefabricated apartment project is narrow and restricted. Hence, a fire explosion can easily be caused when fire occurs. There is no spraying and mechanical smoke exhaust system in the building, and doors and windows are not installed; therefore, natural smoke exhaust conditions were considered in this study.

### Model building

**Figure 3:**
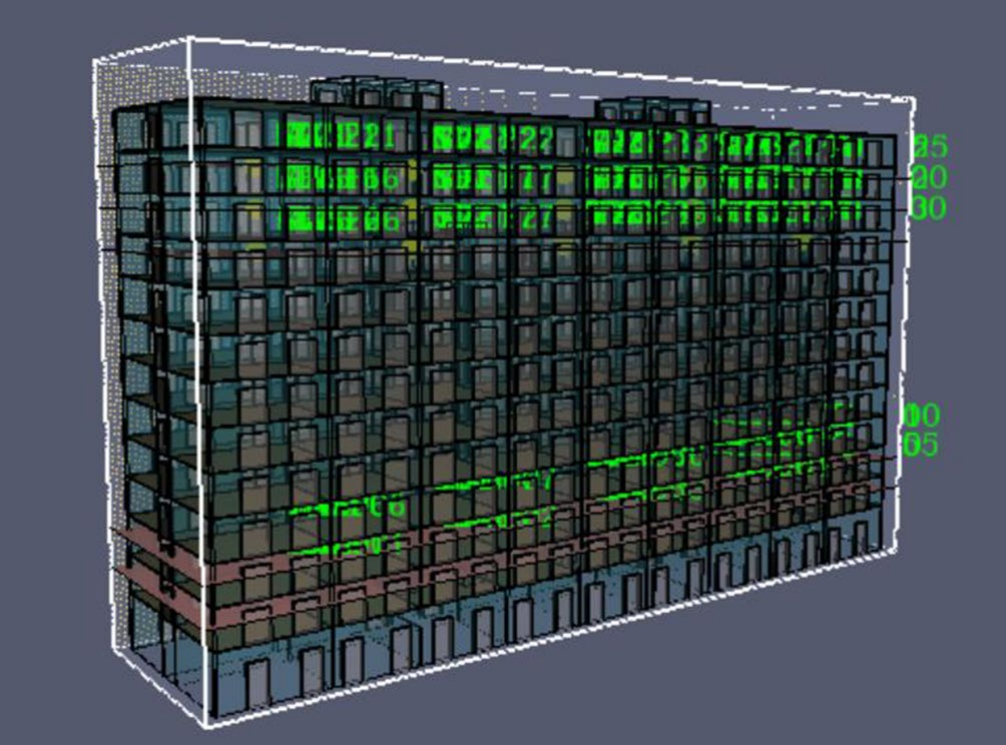
PyroSim model diagram.

**Figure 4:**
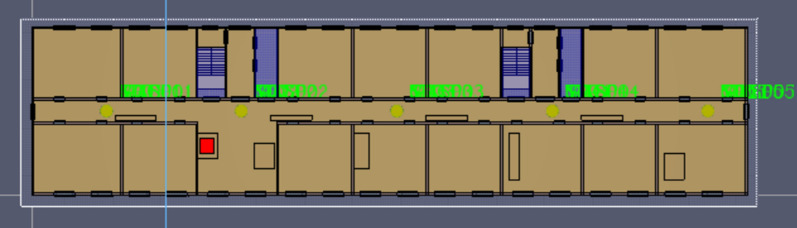
PyroSim Standard layer plan.

A prefabricated apartment building with a height of 3.3 m and a total of 13 floors was considered as the object to be modeled. In this study, the Revit software was used to establish the physical model of fire in a prefabricated building, and the Revit model was then imported into PyroSim. The model renderings and standard floor plans are shown in Figs. 3 and 4. Using the ratio of 1:1, the simulation model was constructed and the model area was divided into grids to simulate a fire scene. If the grid division is too large, it may not accurately calculate and describe the change of the environmental parameters in the fire scene. If the grid division is too small, the accuracy of the simulation calculation will be higher, which may result in excessively long or infeasible calculation. Therefore, by comprehensively considering the calculation accuracy, this study adopted the uniform mesh partition method, and the mesh size was set to 1 m × 1 m × 1 m.

### Fire hazard determination

#### Analysis of factors influencing fire at prefabricated building construction site

According to relevant statistics, most human casualties during a fire are caused by the inhalation of smoke and toxic gas coma before death. When a fire breaks out, the factors that can threaten the safe evacuation of people are the smoke visibility, toxic smoke (mainly CO), and heat radiation from the fire source^23^. The CO concentration, flue gas temperature, flue gas layer height, and flue gas visibility are the main performance parameters. Therefore, the critical values of human tolerance to these parameters should be analyzed to determine whether a fire situation has reached the dangerous state.

1. Smoke layer height

The height of the smoke layer determines whether the smoke will affect the human body. The high-temperature smoke produced in a fire will rise to the upper space of the building under the action of thermal buoyancy. When the smoke encounters the roof of the building, it accumulates and gradually thickens to form a smoke layer. As the smoke continues to rise and the smoke layer continues to thicken, the height of the smoke layer gradually decreases; when the height drops to the height of the fire, harm is caused to the personnel and the evacuation process is greatly affected. Therefore, the smoke layer should be kept above a certain height to ensure the safety of personnel and rapid evacuation to a greater extent. One of the quantitative assessment criteria is that the height of the smoke layer should satisfy the following formula in the process of personnel evacuation^24^.

1 $$H_{S} \ge H_{C} = H_{P} + 0.1H_{B} \left[ {24} \right],$$

where HS is the clear height (m); HC is the critical height (m); Hp is the average height (m) and typically amounts to 1.6 m; HB is the height inside the building (m).

The average height of the investigated prefabricated apartment project was approximately 3.3 m. According to Eq. (1), the critical height HC = 1.6 + 0.1 × 3.3 = 1.93 (m). Therefore, the height of 2.00 m above the ground was considered as the critical height of the smoke layer.

(2) Flue gas temperature

When a fire breaks out, hot smoke is typically produced, and people breathing the overheated air may suffer heat stroke and skin burns. Because thermal radiation data cannot be obtained directly, it is necessary to determine the critical temperature that the human body can withstand by analyzing the fire hazard conditions. The tolerance times of the human body under different conditions are listed in Table 1. The critical value of the selected temperature is 60 °C.

**Table 1:** Limit of human body tolerance to hot air^25^.

Temperature and Humidity	< 60 °C, Water saturation	60 °C, Moisture content < 1%	100 °C, Moisture content < 1%
Tolerance time (min)	> 30	12	1

(3) CO concentration

A large amount of high-temperature smoke is produced during a fire, and the many types of combustibles in buildings, including various toxic and harmful gases and solid particles, among which the CO content is the largest and most harmful to the human body, result in complex fire smoke composition. After CO gas poisoning, carbonyl hemoglobin is produced in the blood and affects the human respiration and nerve reaction. The degrees of harm of different CO concentrations to the human body are listed in Table 2. According to the hazard assessment, the CO concentration of 500 ppm is determined as the critical value for the emergence of danger.

**Table 2:** Degree of harm to human body of different CO concentrations^25^.

CO concentration of flue gas (ppm)	Time (min)	Degree of hazard
200	120–180	Mild headache and fatigue
400	60–120	Secondary headaches
800	45	Unconsciousness, vomiting
1600	20	Headache, dizziness, nausea
3200	10–15	Death

(4) Smoke visibility

The visibility of flue gas determines people’s capability of assessing the surrounding environment and recognizing the evacuation passage and safety exit, the perception and decision-making process of trapped personnel, the escape time, and other factors affecting the efficiency of an emergency evacuation. Low visibility results from the blocking of light by the solid particles in flue gas. In a low-visibility environment, the suspended solid particles stimulate the eyes, make it difficult for trapped people to accurately assess the surrounding environment, and reduce people’s ability to recognize the evacuation channels and safety exits, which results in perceptual decision-making mistakes and missing the optimal escape time. The Australian “Fire Engineer Guide” specifies the critical value of visibility for spaces with different size. The critical value of visibility is 10 m in a large space and 5 m in a small space^26^. The investigated prefabricated apartment project has small space; therefore, the smoke visibility index is 5 m.

#### Fire hazard determination conditions

Through the analysis of the above-mentioned factors influencing the safety of personnel evacuation, the temperature, CO concentration, and visibility conditions in the fire scene should be set to ensure the safe evacuation of construction personnel. This study set the fire risk assessment conditions of the prefabricated apartment project as follows:

1. Temperature assessment conditions: a smoke layer temperature exceeding 60 °C at a height of 2.00 m above the ground is considered as a fire danger state.
2. CO concentration assessment conditions: a CO concentration greater than 500 ppm at a height of 2.00 m above the ground is considered as a fire danger state;
3. Visibility assessment conditions: if the visibility of the smoke layer at 2.00 m above the ground is less than 5 m, it is considered that the fire has reached the dangerous state.

### Fire scene setup

**Figure 5:**
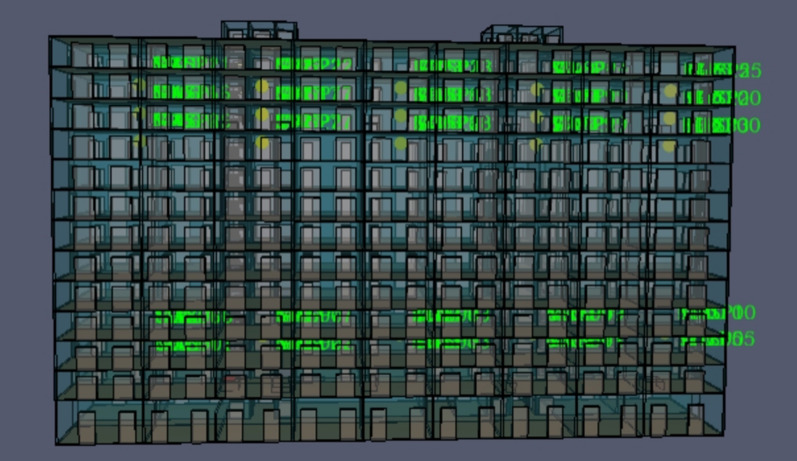
Sensor relative position diagram (the circle marks the location of the fire source).

Initial simulation environment: the flow field in the room is static, the temperature is 20 °C, and the pressure is standard atmospheric pressure. A building fire has the characteristics of fast spread, difficult management, and high rescue difficulty. Because the prefabricated building is under construction, there is no automatic fire extinguishing system, smoke exhaust system, or other similar equipment, and the construction situation of each part must be analyzed simultaneously. Two ignition sources were set in the numerical simulation study. The first fire was located in the left corridor on the 2nd floor of the lower level, and the second fire was located in the left corridor on the 11th floor of the higher level. To monitor the spread of office building fire and the visibility and temperature change characteristics of the escape routes, several temperature sensors, CO concentration detectors, smoke layer height detectors, and visibility detectors were set in the fire layer and upper and lower adjacent layers. The height is 2 m above the ground of this layer, as shown in Fig. 5.

### Analysis of fire simulation results

#### Fire smoke spread analysis

**Figure 6:**
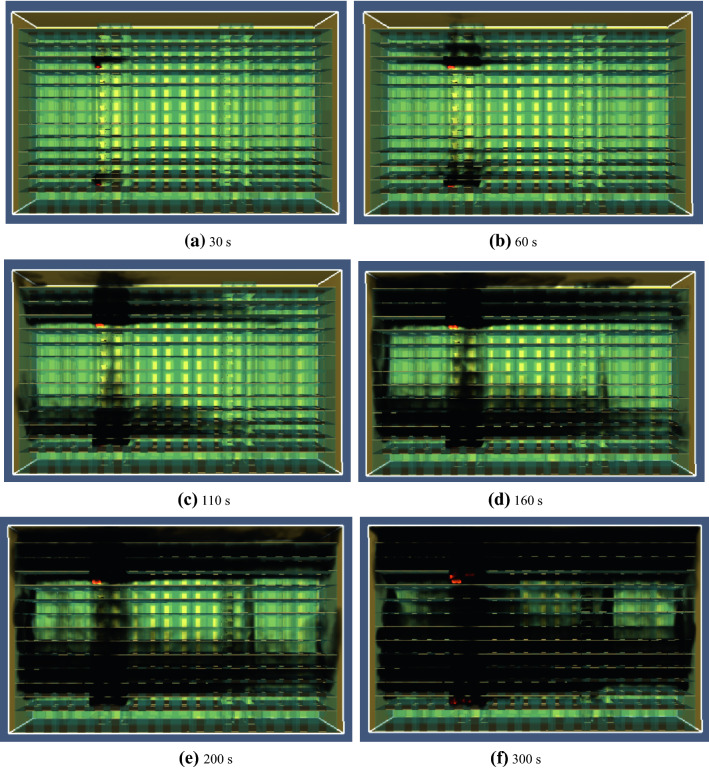
Chart of smoke spread.

Figure 6 shows the process of simulating the spread and filling of smoke in an office building after a fire. As can be seen from the simulation results presented in Fig. 6a–f, As can be seen from the simulation results in Fig. 6a–f, smoke mainly collects around the fire source in the initial stage of fire combustion when the fire occurs 30 s. Sixty seconds after the fire has broken out, the hall on the left side of the 2nd floor and 11th floor is filled with smoke spreading rapidly upward through the stairwell and elevator shaft. At 110 s after the onset of fire, the smoke from the fire source on the 11th floor spreads to the top floor, and the smoke from the fire source on the second floor spreads to the talent apartment through the west window. At this time, the smoke does not fill the entire corridor on the second floor. As the fire develops, the smoke continues to spread, and the smoke concentration gradually increases. At t = 160 s, the east, middle, and west areas of the 2nd and 11th floors are completely filled with smoke. At 200 s after the fire occurs, the floors adjacent to the fire floor are completely filled with smoke. Three hundred seconds after the fire has broken out, the smoke continues to spread and all floors except the first floor are filled with smoke. Approximately 75% of the entire office building is filled with smoke.

#### Temperature change analysis

**Figure 7:**
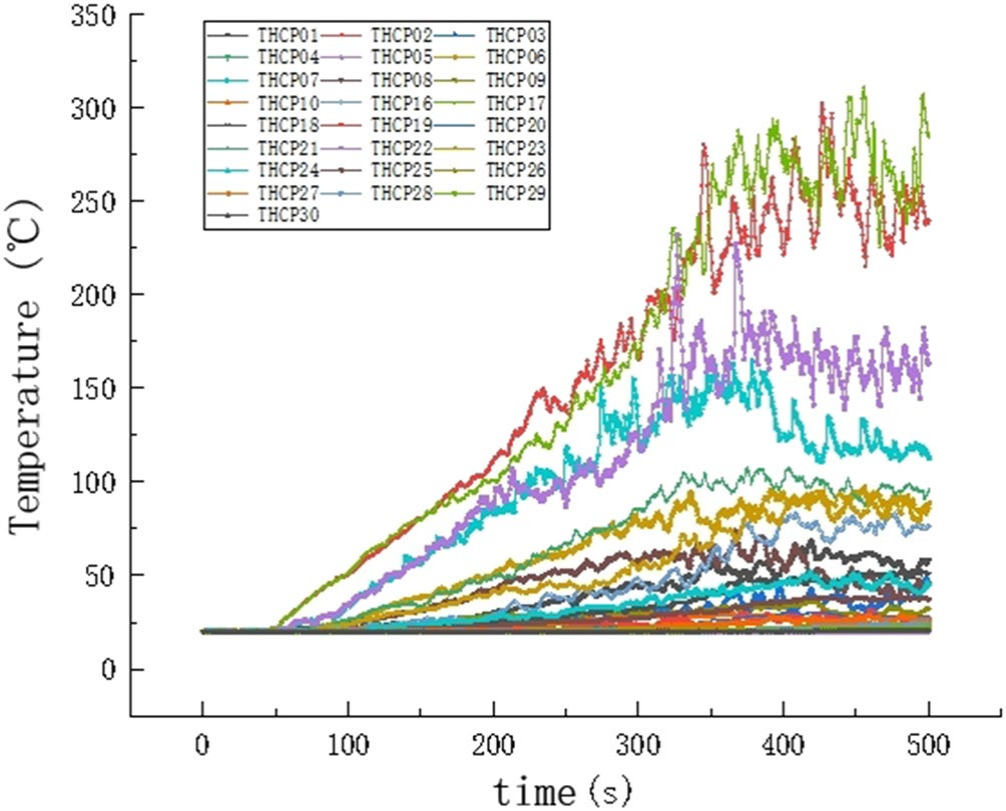
Temperature curve.

**Figure 8:**
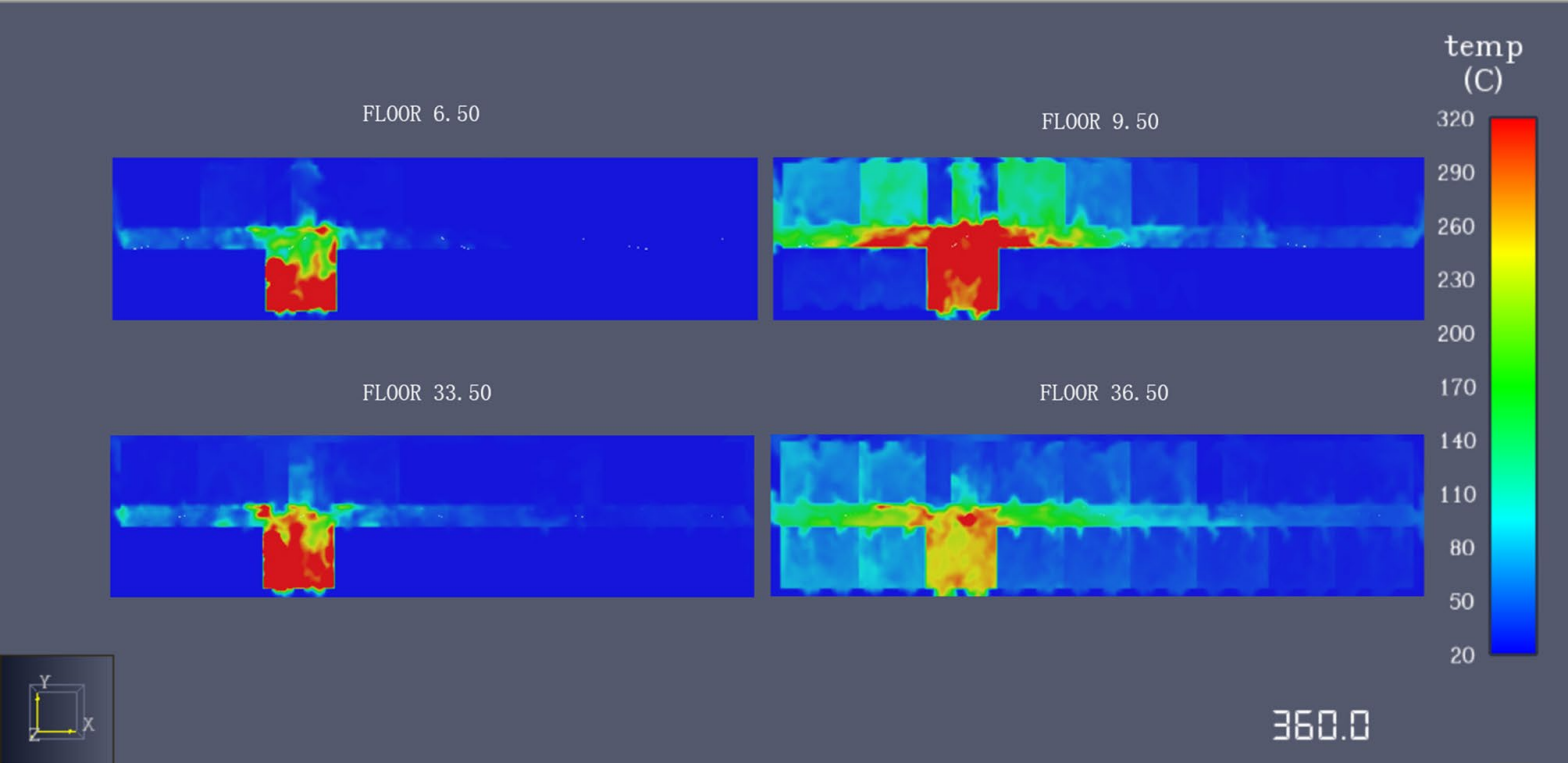
Slice diagram of temperature change.

The temperature change curve and the temperature change slice diagram is drawn in Figs. 7 and 8. According to the temperature change data obtained by the temperature sensor. By analyzing the temperature change curve, the following conclusions are obtained: (1) the fire location exhibits approximately the same change trend, which reveals that, after the initial stage of fire combustion, the temperatures soars to the maximum temperature fluctuation and then declines. With the spread of the fire, the temperature began to rise after reaching the lowest point, and the temperature tended to be stable with the development of time. (2) When fire occurs, the temperature at the fire source linearly increases, which can easily cause severe threats to the life and safety of personnel close to the fire source. Hence, it is very important for people to escape the danger immediately and stay away from the fire source. (3) The temperature change trend at the exit of the stairs is the same and the temperature change is relatively slow; as the distance to the fire decreases, the temperature change becomes faster. (4) According to the above discussion, the ambient temperature limit of the human body is approximately 60 °C, and the data of six sensors exceed 60 °C within 350 s.

#### CO concentration change analysis

**Figure 9:**
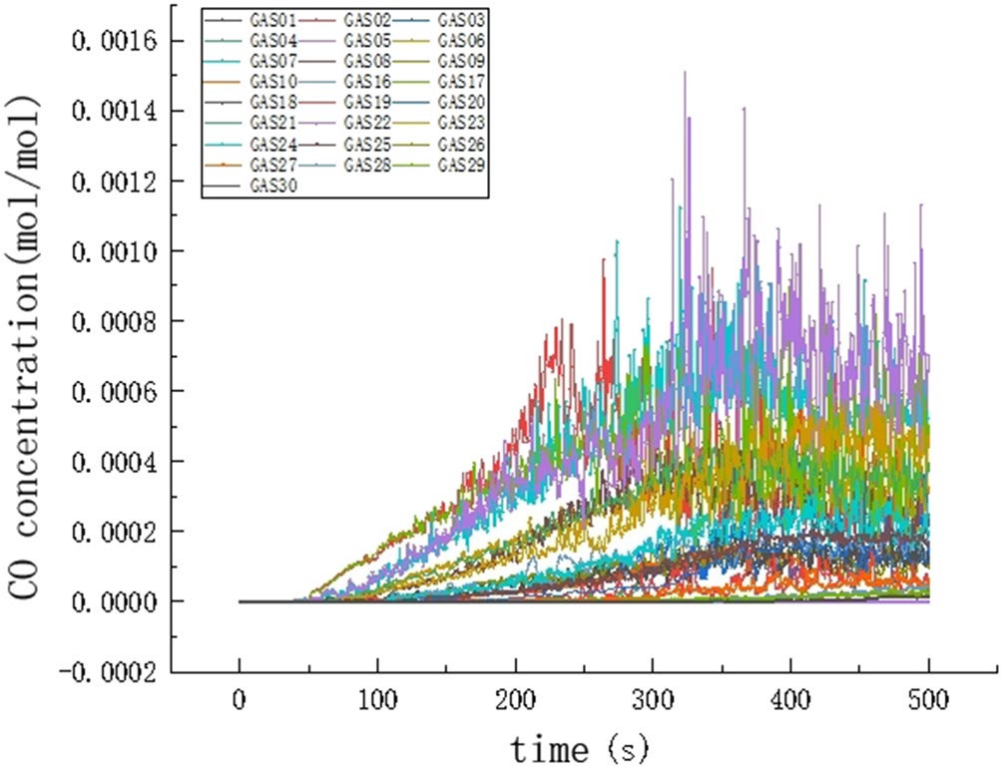
Curve of CO concentration change.

**Figure 10:**
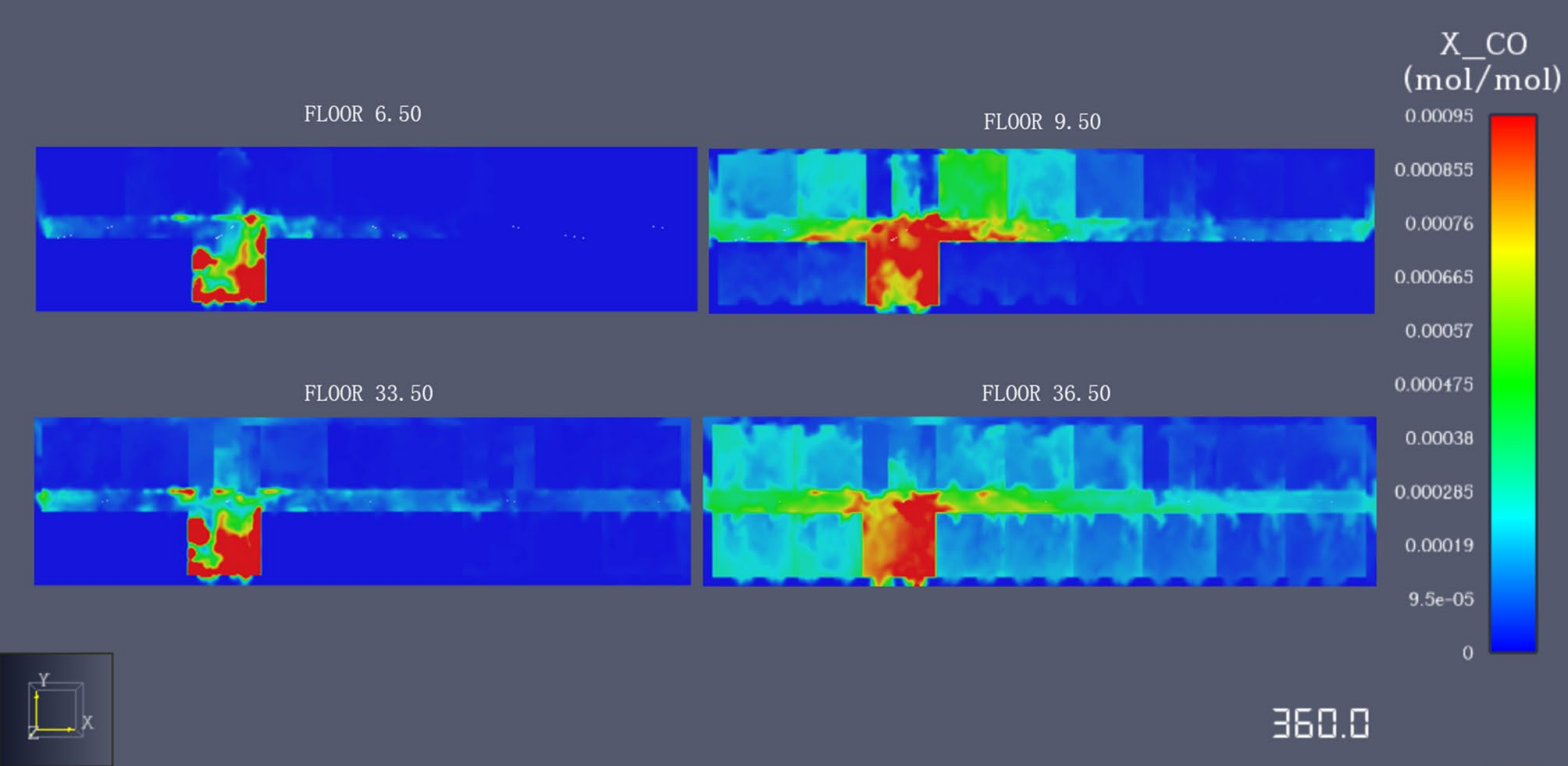
Section diagram of CO concentration change.

Under the action of plume, the buoyancy of the flue gas increases and the flue gas rapidly rises to the ceiling. The produced flue gas jets to the ceiling and rapidly spreads to both sides of the corridor along the ceiling. The carbon monoxide concentration is maximum at the detection point farthest from the fire source, where a large amount of flue gas has accumulated. Under the positive chimney effect, hot flue gas is gradually injected into each floor over time. The flue gas in the stairwell is affected by the positive chimney effect, and the temperature of the flue gas increases; therefore, the flue gas produces greater buoyancy. Under these conditions, a large number of high temperature flue gas moves to the upper layer. However, during the movement, the flue gas is hampered by the staircase structure, and a large amount of high temperature flue gas accumulates at the stairs, which leads to a further increase in the flue gas temperature. Hence, the flue gas produces stronger buoyancy, and the temperature flue gas action is high until the gas moves to the top of the stairs. Additionally, at 11 layer detection points, the carbon monoxide concentration is higher relative to other floors, and thus more dangerous, as shown in Figs. 9 and 10. When the height of the smoke layer is smaller than the height of the human eye, the carbon monoxide concentration at all detection points does not reach the critical value during the simulation time, and the carbon monoxide is not harmful to humans.

#### Visibility change analysis

**Figure 11:**
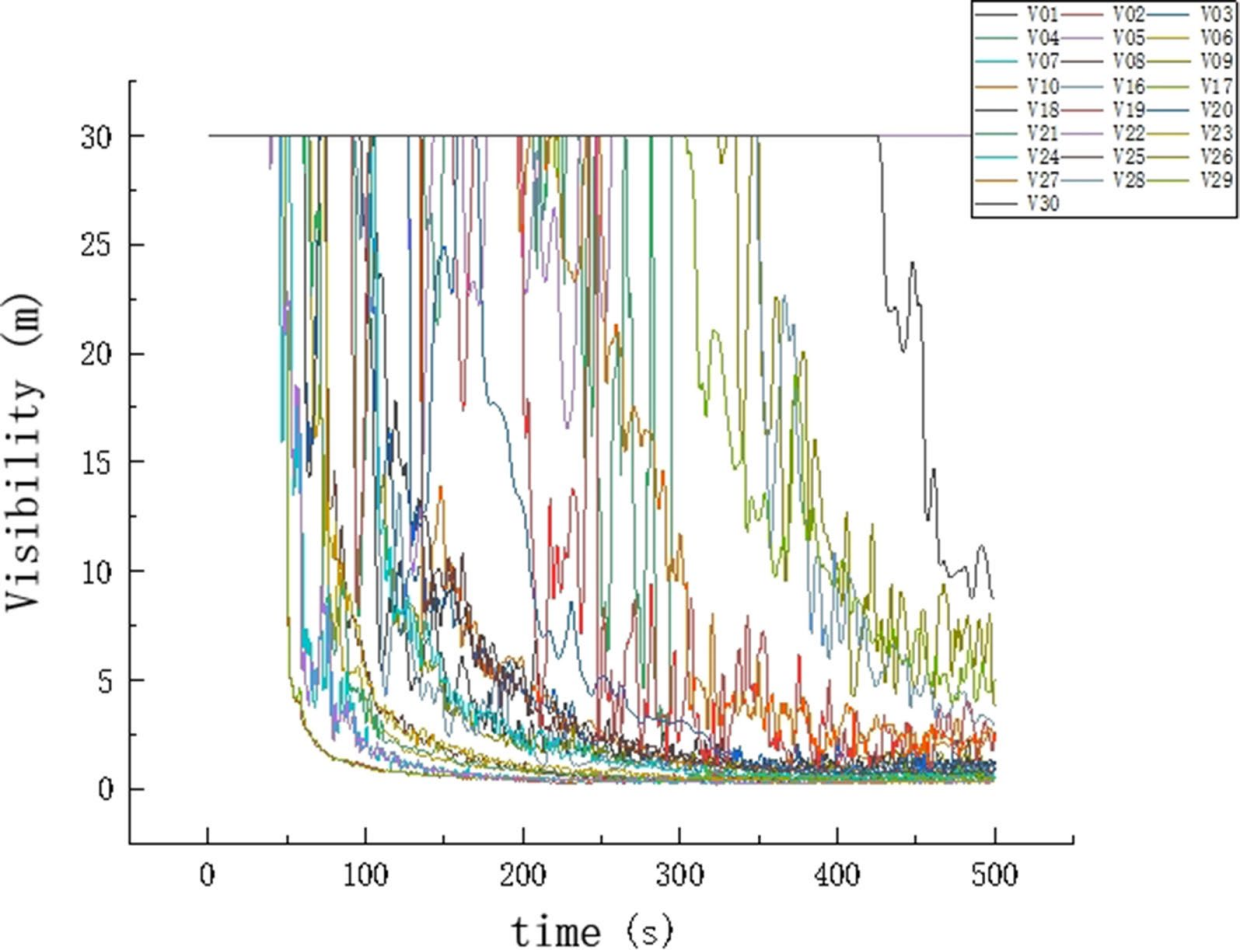
Visibility change chart.

**Figure 12:**
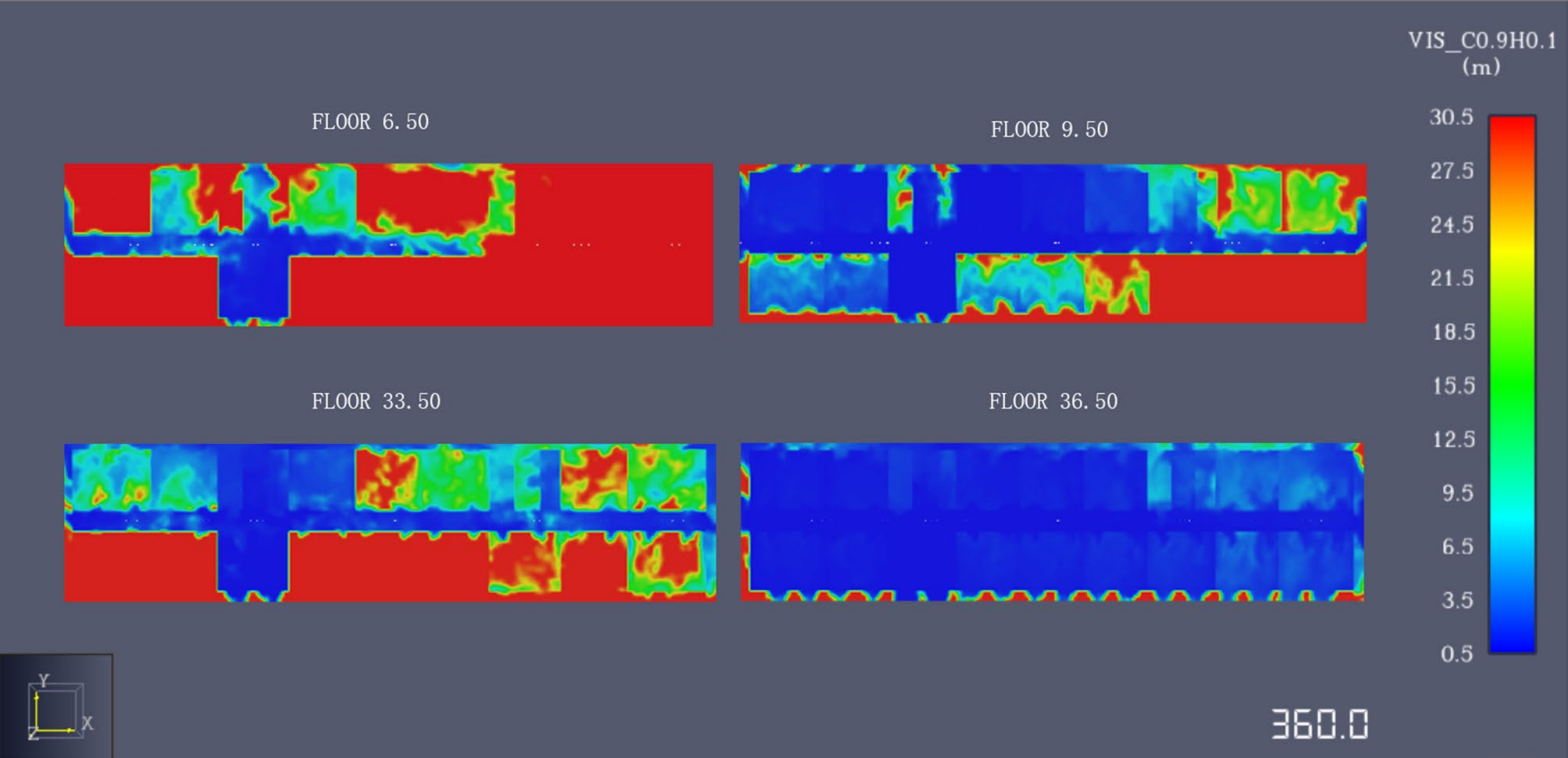
Slice diagram of visibility changes.

In the process of combustion, solid and liquid particles are produced in large numbers, which greatly affects the visibility of the fire site and produces great resistance for the emergency evacuation of personnel and the smooth extinguishing of the fire. Therefore, this study installed visibility sensors at the same positions as the temperature sensors. The visibility curve was drawn according to the sensor data. As shown in Figs. 11 and 12, in the early stage of the fire, the visibility of the floor was approximately 30 m and decreased exponentially as smoke filled the office building with the spread of fire. Owing to the impact of the fire source on the 11th floor, the visibility of the sensor decreased very early. The visibility dropped to 5 m in 50 s and reached the danger point for casualties. At approximately 400 s, the data collected by all visibility sensors reveal that the visibility at each exit was less than 5 m. The optimal escape time elapses after 400 s.

## Establishment of numerical model and emergency evacuation simulation

### Evacuation software contrast with Pathfinder software introduction

At present, several types of evacuation simulation software are widely used (Pathfinder, FDS + EVAC, Building Exodus, Steps, Simulex, and so on) and their application scopes are listed in Table 3. Pathfinder is an intelligent evacuation evaluation software 21 that is simple to use, visually intuitive, and convenient to apply. Additionally, Pathfinder is suitable for large complex buildings and has the following advantages:

1. The software contains its own internal modeling system, and additional formats can be used through the import of modeling files.
2. The model has fast calculation speed and can handle the evaluation and analysis of large complex buildings for personnel evacuation research.
3. The software comprehensively considers the parameters affecting personnel evacuation and can more accurately calculate the personnel evacuation situation at a specific building in a specific scene.
4. The software can partition the building model and simultaneously determine the evacuation process and escape route for people in designated positions on different floors.

**Table 3:** Common evacuation simulation software and its applicable scope.

Software brand	Scope of application
Building EXODUS	Airports, stations and other large Spaces and buildings with large escape crowd
FDS + Evac	Labor-intensive factories, stadiums and other public places and large vehicles
Pathfinder	Suitable for large buildings
Simulex	Suitable for large, complex geometrical buildings with multiple floors and staircases
STEPS	Various building types and vehicles

Therefore, this study selected the Pathfinder software to numerically simulate the process of evacuating Prefabricated building construction site staff.

Pathfinder is an emulator based on personnel evacuation and movement simulation^22^. The software is a three-dimensional (3D) mesh model that simulates evacuation under normal and emergency situations. The Pathfinder software can define the parameters of pedestrians. After the evacuation begins, the pedestrians will respond to changes in the evacuation environment and select the optimal evacuation path^23^.

### Establishment of emergency evacuation environment

**Figure 13:**
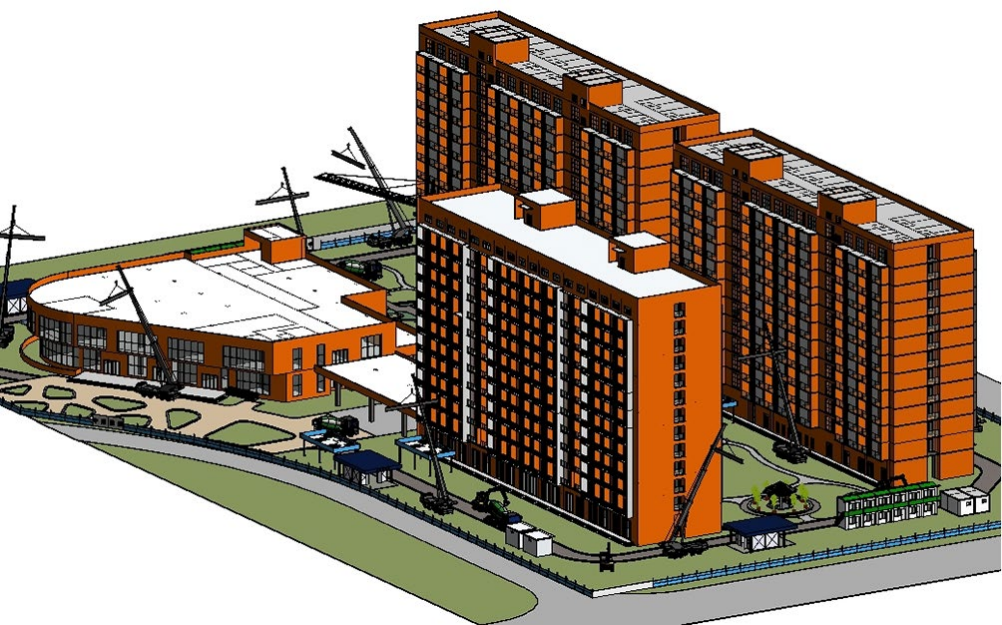
BIM security information model.

**Figure 14:**
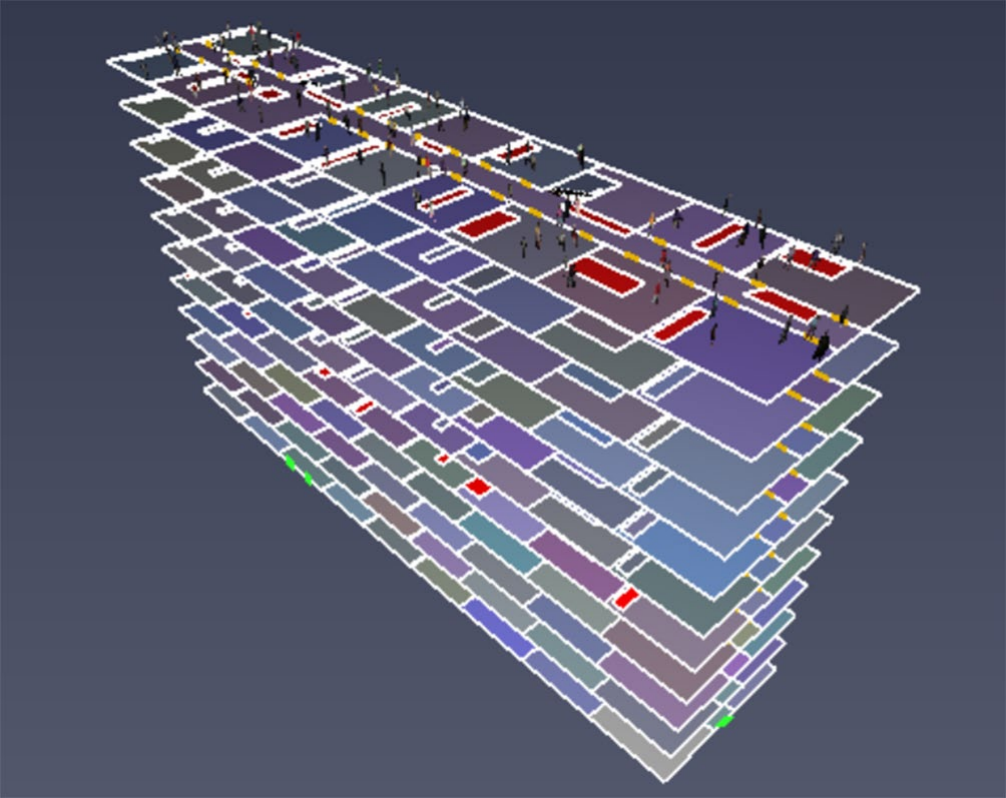
Pathfinder model of safe emergency evacuation.

To satisfy the requirements of the evacuation simulation, this study converted the safety information model of an assembly-type talent apartment project into the DXF format and imported it into Pathfinder software. The model was simplified, and the floor, stairs, doors, other components, and evacuation routes were identified. The BIM safety information model, Pathfinder evacuation model, and personnel distribution in an evacuation scenario are shown in Figs. 13 and 14.

### Setting of emergency evacuation barriers

**Figure 15:**
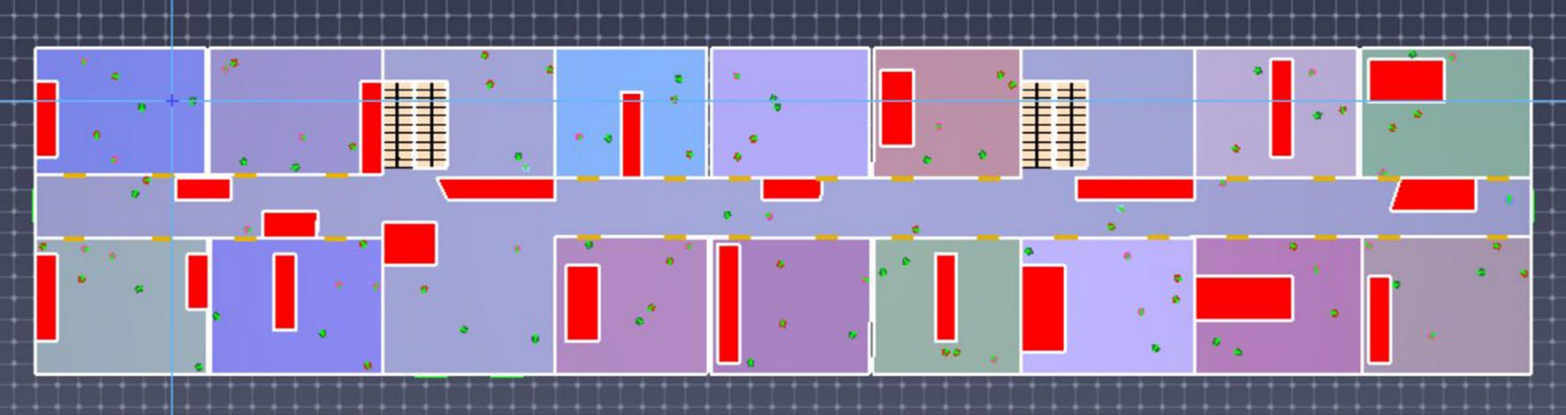
Obstacle setup in Pathfinder emergency evacuation model.

Prefabricated construction is complex and ever-changing, and the construction process, construction environment, and change of construction plane greatly influence the speed of personnel evacuation, evacuation zone, and evacuation path. In this paper, obstacles such as prefabricated components and construction instruments are set up according to the on-site construction status of prefabricated buildings, as shown in Fig. 15.

### Set emergency evacuation parameters

To establish a safe evacuation simulation, the behavior of people trapped at a specific location and in a certain environment must be simulated. The evacuation personnel, evacuation location, and evacuation environmental conditions are the main considerations in the simulation of safe evacuation^27^. Owing to the particularity of the prefabricated construction industry, the on-site construction personnel consist of basic assembly technology workers and manual laborers, and these laborers can be divided into four categories: young men, middle-aged men, and young and middle-aged women. Different types of personnel escape velocity have a larger difference, and the evacuation simulation software will conduct evacuation simulation according to the set personnel attribute characteristics. Additionally, the working face of the main body of the assembly building in the construction stage can be divided into two types: floor plane and stair plane. The speed of different types of personnel on different working faces must be set before the evacuation simulation.

1. Set staff signs

The parameters and proportions of the physical personnel attributes are listed in Table 4.

**Table 4:** Physical sign parameters and proportions of construction personnel.

Personnel category	Young men	Young women	Middle-aged men	Middle-aged women
Shoulder breadth (cm)	41.0	38.0	41.9	39.5
Thick chest (cm)	25.8	25.7	26.4	26.6
Proportion (%)	40.7	18.5	29.5	11.3

(2) Set staff speed

According to the investigation and analysis of the evacuation speed of construction personnel on the assembly building site^1^, the personnel speed was set as presented in Table 5.

**Table 5:** Evacuation speed of different types of personnel on different working surfaces.

Working plane	Category			
	Young men (m/s)	Young women (m/s)	Middle-aged men (m/s)	Middle-aged women (m/s)
Plane	1.95	1.79	1.84	1.68
Stair surface	1.25	0.98	1.19	1.03

(3) Behavioral pattern settings

The behavioral modes of Pathfinder software are the SFPE mode and steering mode. In the case of an emergency, site construction personnel exhibit three behavioral modes: the shortest distance behavioral mode, the consistent in-and-out behavioral mode, and the complete conformity mode. According to the data analysis, approximately 20% of people will wait in line at the same place, while approximately 50% of people will choose another evacuation route. The steering mode can control the evacuation of personnel by combining path planning, the guidance mechanism, and collision processing. Therefore, this study selected the steering mode of emergency evacuation.

### Analysis of evacuation simulation results

The simulation of the safe evacuation of construction personnel was carried out based on the determination of the above parameters. As shown in Fig. 15, a large number of prefabricated components, construction tools, and other items were piled up on the construction site of the prefabricated building, and many items were stacked around the evacuation corridor and stairs. When an emergency occurs, these items are likely to block people’s evacuation path, and the evacuation speed will be greatly reduced. Therefore, the number and location of stacked items on the assembly building construction site and the number of people on each floor were the focus of the emergency evacuation. Because there are different construction stages and the flow of the construction site personnel is relatively complex, the number of evacuees at the construction site could not be accurately determined in this study. Based on the principles of the most unfavorable apartment and taking the construction plane model of the 11th floor of the higher fire floor of building F02# of talent apartment project as reference, and according to the actual personnel investment situation of the construction site of prefabricated building, the simulated site construction personnel was set to 100 people, who were assumed to be randomly distributed in different working areas on the 11th floor. Thus, the emergency evacuation simulation was carried out.

1. Results of evacuation simulation

**Figure 16:**
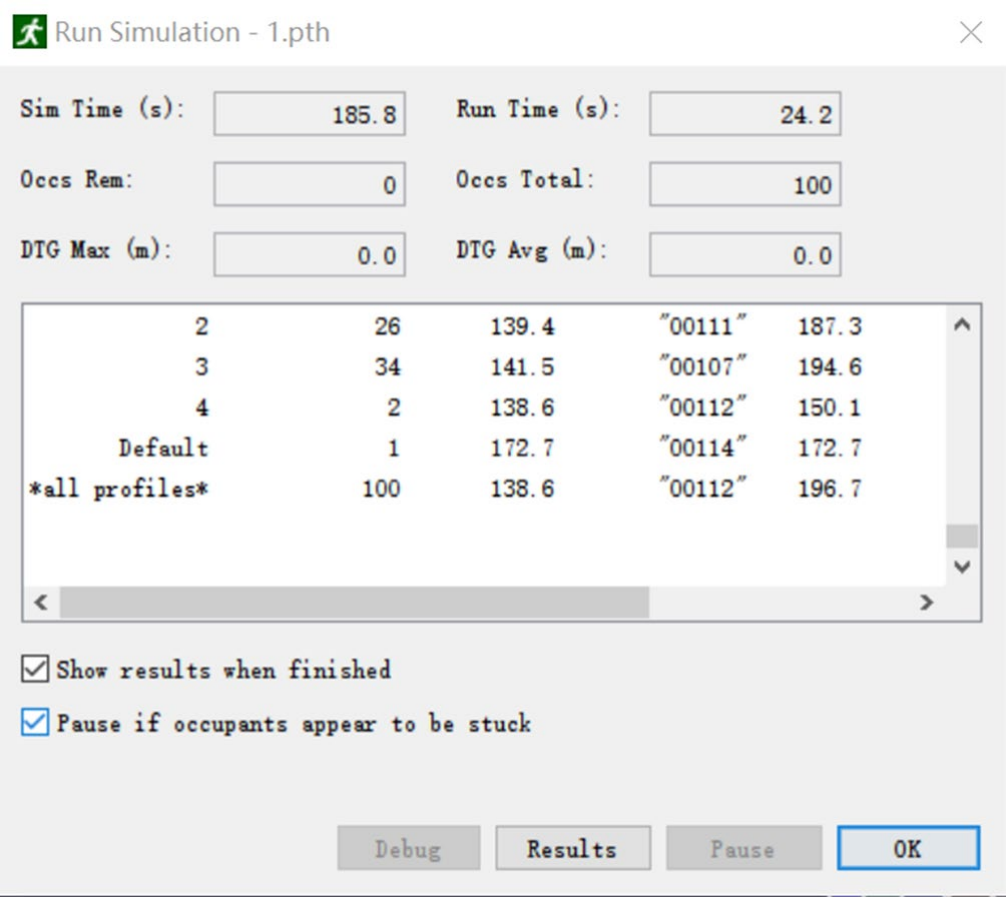
Total evacuation time.

**Figure 17:**
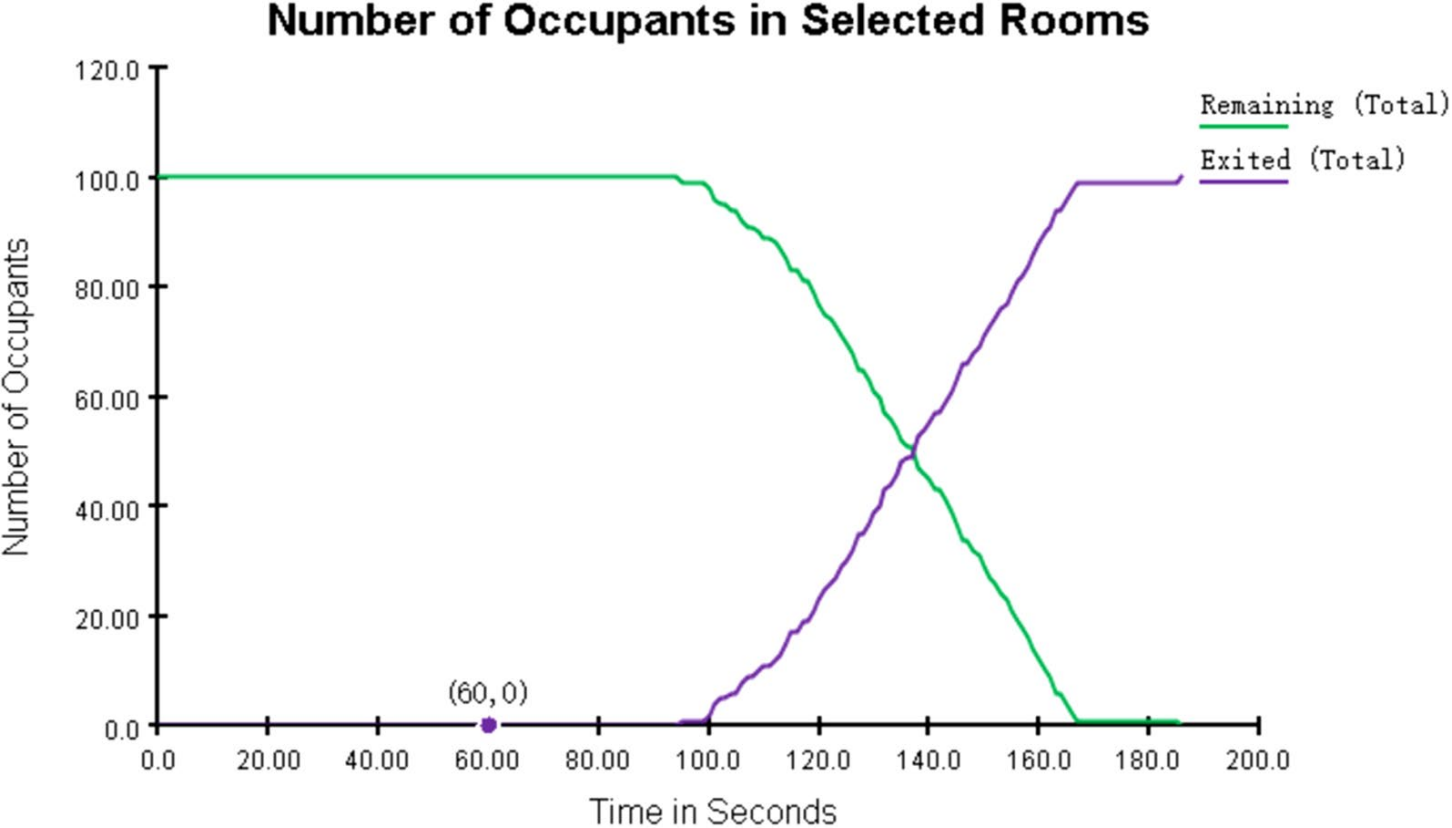
Evacuation time-number curve.

**Figure 18:**
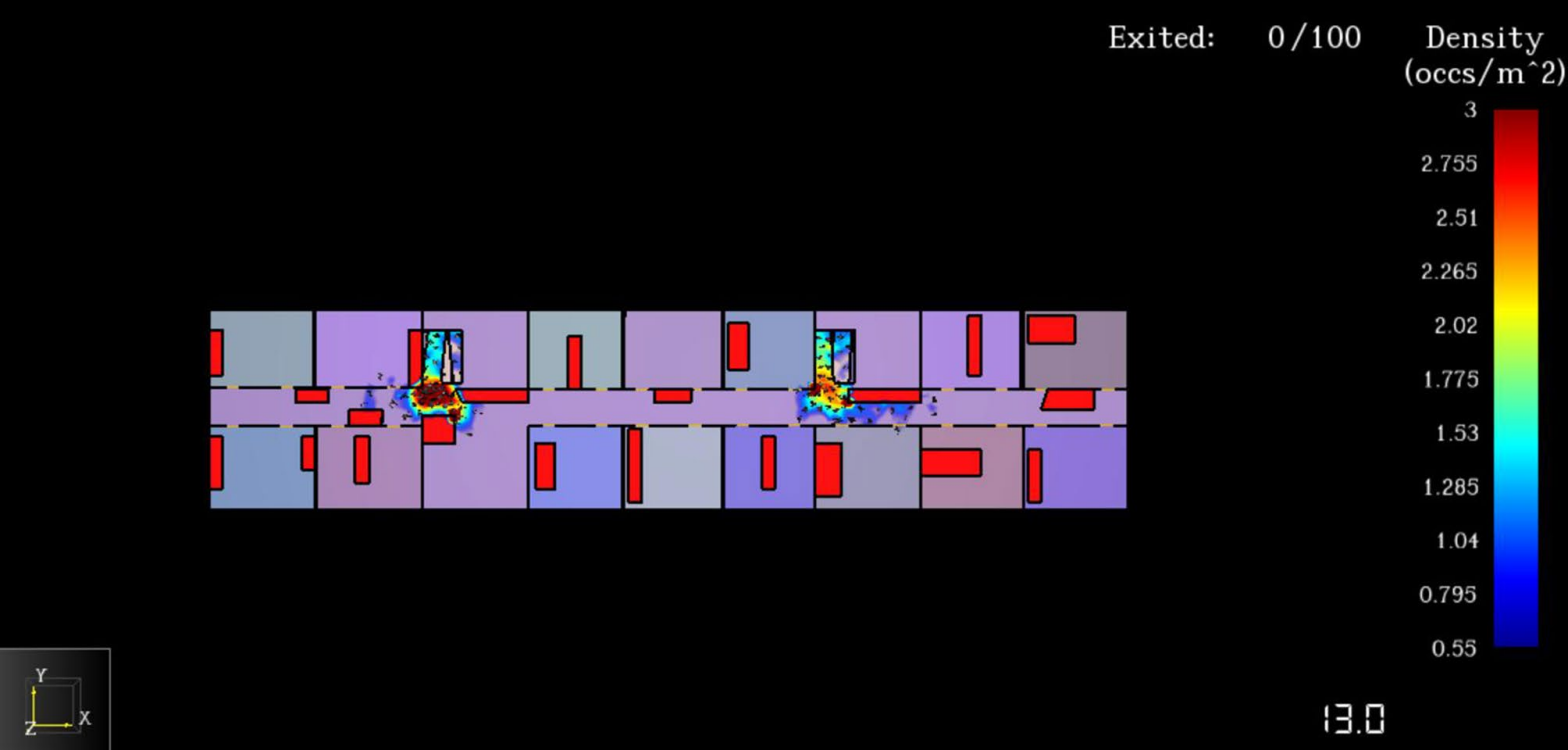
Personnel density map at evacuation time of 13.0 s.

The results of the evacuation simulation are shown in Figs. 16 and 17. A total of 100 on-site construction personnel were evacuated in 185.8 s. Figure 18 shows the personnel density diagram at the evacuation time of 13.0 s. As can be seen, the evacuation corridor and stairway are the areas with the highest personnel density, where evacuation corridor and stairway traffic jams can easily occur. However, there are two evacuation staircases on the east and west sides of the construction site of the talent apartment project. Therefore, prefabricated materials and mechanical appliances and other items must be removed from the evacuation corridors and staircases.

(2) Safety determination

When fire breaks out in a building, the safe evacuation of on-site personnel mainly depends on the time required for safe evacuation (T_RSET_) and the available time for safe evacuation (T_ASET_). The on-site personnel can be safely evacuated only when the following formula is satisfied, otherwise the scheme should be adjusted.

2 $${\text{Required}}\,{\text{time}}\,{\text{for}}\,{\text{safe}}\,{\text{evacuation }}\left( {{\text{T}}_{{{\text{REST}}}} } \right) \, { \leqq }{\text{ Available}}\,{\text{time}}\,{\text{for}}\,{\text{safe}}\,{\text{evacuation }}\left( {{\text{T}}_{{{\text{ASET}}}} } \right).$$

Here, (T_REST_) refers to the time needed for all on-site personnel to be evacuated to safety from the moment when the emergency is discovered; (T_ASET_) refers to the time limit after which the on-site personnel are exposed to the risk of external accidents after the occurrence of a fire.

3 $${\text{Required}}\,{\text{time}}\,{\text{for}}\,{\text{safe}}\,{\text{evacuation }}\left( {{\text{T}}_{{{\text{REST}}}} } \right) \, = {\text{ Alarm}}\,{\text{time }}\left( {{\text{T}}_{{{\text{alarm}}}} } \right) \, + {\text{ Response}}\,{\text{time }}\left( {{\text{T}}_{{{\text{pre}}}} } \right) \, + {\text{ Evacuation}}\,{\text{time }}\left( {{\text{T}}_{{{\text{move}}}} } \right).$$

In Eq. (3), T_move_ refers to the time required to evacuate all on-site construction personnel, and is jointly determined by human attributes, psychological factors, environmental characteristics, and other factors. Therefore, T_move_ is highly uncertain. Hence, the evacuation time in domestic and overseas fire control design must be multiplied by a coefficient of 1.5–2. Additionally, T_alarm_ refers to the time when some on-site construction personnel discover the accident and notify all other construction personnel. The case selected in this study is the main stage of the assembly building construction site. Because there are few obstacles around the building and the visibility is better, it is assumed that on-site construction personnel can quickly identify the abnormal situation when a fire breaks out. Therefore, T_alarm_ was set to 60 s. Finally, T_pre_ refers to the response time of the field construction personnel after receiving instructions pertaining to the danger. Because a safety risk warning system and monitoring system were installed at the construction site of the simulation project, the response time was considered to be relatively short, and was set to 120 s.

Safety determination:

Let us consider that T_REST_ = T_alarm_ + T_pre_ + 1.5 × T_move_ = 60 + 120 + 1.5 × 185.5 = 398.7 s. According to the above formula, the available time for safe evacuation is 400 s. Moreover, the average shock time of buildings domestically and abroad is 5–7 min^28^; the shock time (T_ASET_) was set to 6 min in this study. The required time for safe evacuation (T_REST_) > Available safe evacuation time (T_ASET_). Therefore, the safety assessment failed, and all personnel could not be evacuated to safety.

(3) Optimization of construction site layout and number of construction personnel

**Figure 19:**
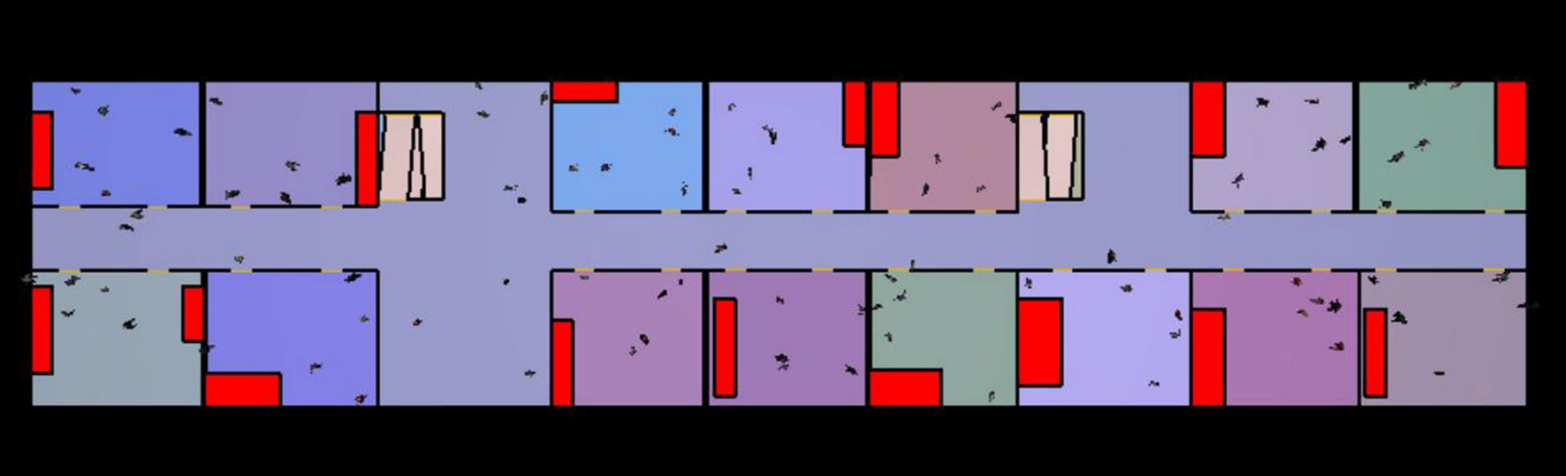
Optimized stacking of building materials.

The results of the first emergency evacuation were analyzed. First, the items stacked at the evacuation stairs and corridors affected the speed of personnel movement. To optimize the prefabricated materials and mechanical equipment, the obstacle positions must be optimized and specific operations must be carried out to remove items from the evacuation paths. Additionally, the obstacles blocking the indoor corridors and evacuation stairs should be transferred inside the room as far as possible and situated at the corner or on the side. Secondly, the excessive number of on-site personnel affected the overall evacuation speed. Therefore, the simulated number of the second emergency evacuation was reduced to 80 people. The optimized stacking of on-site items and personnel settings are shown in Fig. 19. A second emergency evacuation simulation was conducted with the optimized settings.

**Figure 20:**
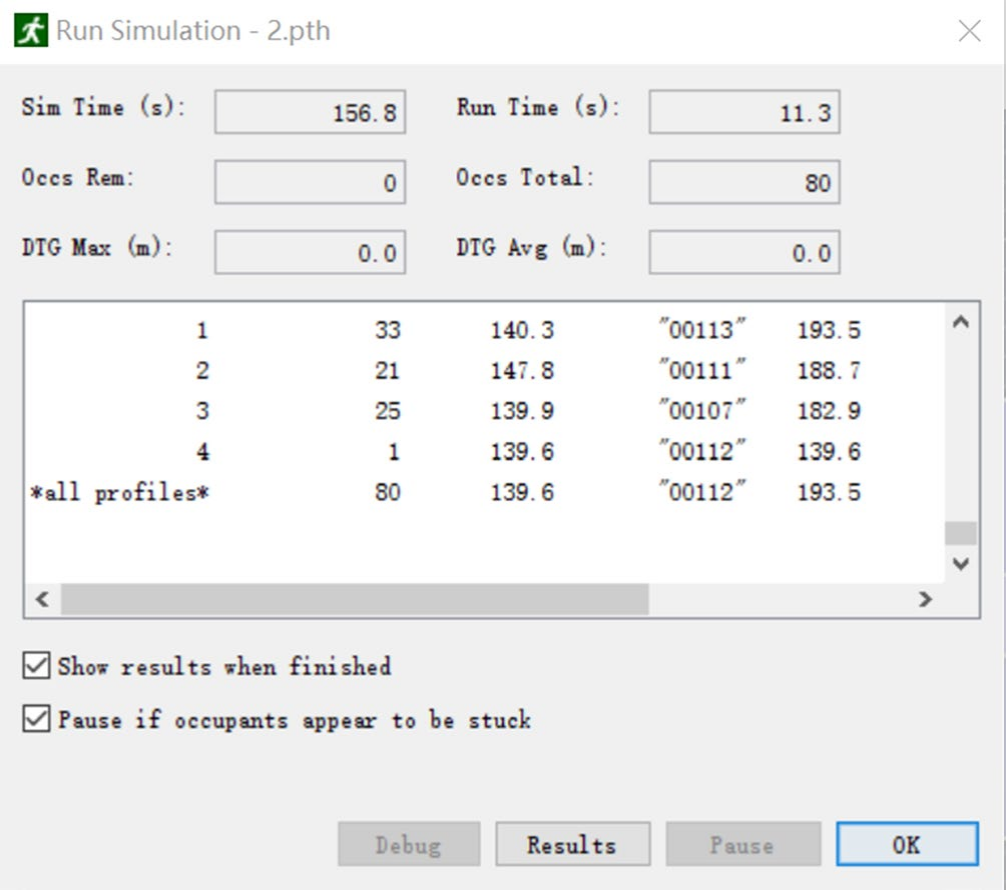
Total evacuation time (second simulation).

**Figure 21:**
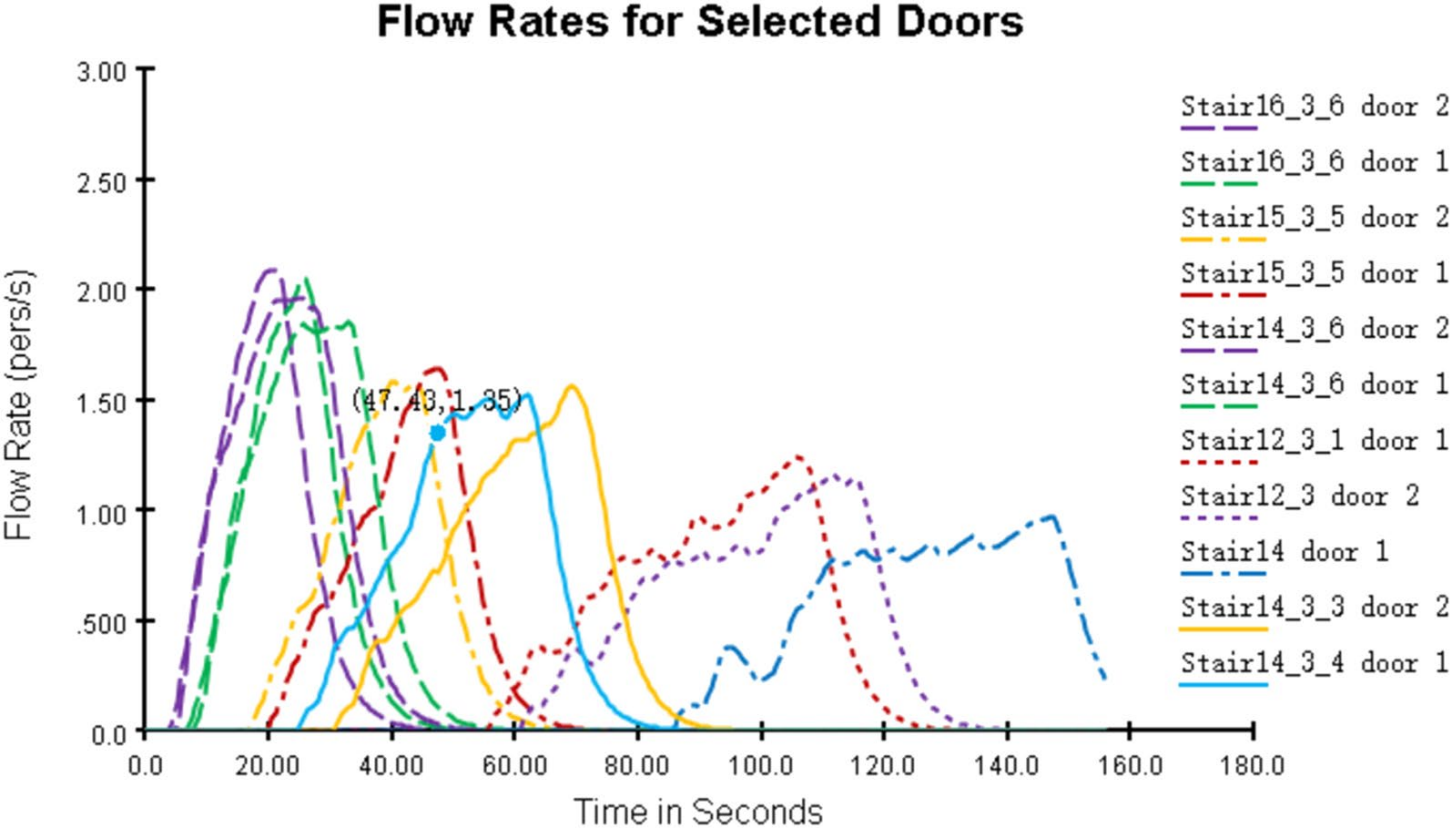
Changes in flow of people at each exit.

As shown in Fig. 20, the total time used in this simulation was 156.8 s. The time required for safe evacuation is T_REST_ = T_alarm_ + T_pre_ + 1.5 × T_move_ = 60 + 60 + 1.5 × 156.8 s = 355.2 s, and T_REST_ = 355.2 s < T_ASET_ = 360.0 s, which satisfies the code requirements for the design of building fire prevention measures. Figure 21 shows the efficiency of each staircase and entrance.

## Conclusion

The following conclusions were drawn from this study:

1. When a fire occurs, the temperature variation at the fire source is the most obvious variation, and the temperature variation trend is approximately the same. The smoke rising through the stairwells and elevator shafts to the 13th floor is the main factor contributing to the filling of the middle area of the 13th floor with smoke.
2. Based on a prefabricated apartment, the fire simulation and emergency evacuation simulation are studied. Through in-depth analysis, it was found that the evacuation efficiency is related to the personnel density, relative position of personnel, and degree of evacuation congestion. Severe detention and congestion in the stairwell were the main reasons affecting the evacuation efficiency, and should be considered as the primary safety hazards in the prevention of actual fire emergencies.
3. Visibility variation features can guide the evacuees and assist the rescue effort. In the numerical simulation conducted in this study, the optimal emergency rescue entrance was the east escape exit.
4. The total time of the first emergency evacuation was 185.8 s. By analyzing the data collected by the temperature sensor, CO concentration sensor, and visibility sensor in the scene, it was found that the visibility and shock time are the key factors that negatively affect the evacuation efficiency. At 400 s, the visibility of the exit of the prefabricated apartment was less than 5 m. The building crash time was 360 s, which reaches the danger point for casualties. When the time required for safe evacuation (T_REST_) > available time for safe evacuation (T_ASET_), the safety assessment fails, and the on-site personnel cannot be completely evacuated to safety.
5. The evacuation time can be effectively reduced by reasonably planning the position where assembly construction materials, equipment, and other items are stacked to reduce the loading of the construction plane, ensure that the evacuation stairs are unimpeded, and actively provide evacuation guidance. The results of the second simulation reveal that the safe evacuation time (T_REST_) is 156.8 s. The time required for safe evacuation (T_REST_) < available time for safe evacuation (T_ASET_) is in line with the requirements of the building fire prevention design code.
6. This study considered a prefabricated apartment and used the PyroSim and Pathfinder software to numerically simulate a fire and emergency evacuation, so as to determine how to effectively evacuate on-site personnel to safety during a fire emergency. The obtained results reveal that simulation technology has good potential for use in high-rise building fire simulation and evacuation research. This study considered a fixed fire point to investigate the spread of fire and the evacuation of personnel, and can provide a foundation for future work considering multiple fire points and different obstacle distributions to obtain more significant results.

## Data Availability

The data that support the findings of this study are available from the corresponding author upon reasonable request.
